# Insertion of a foldable hydrophobic IOL through the trabeculectomy fistula in cases with Microincision cataract surgery combined with trabeculectomy

**DOI:** 10.1186/1471-2415-6-14

**Published:** 2006-03-19

**Authors:** Tanuj Dada, Rajamani Muralidhar, Harinder S Sethi

**Affiliations:** 1 Centre for Ophthalmic Sciences, All India Institute of Medical Sciences, New Delhi 110029, India

## Abstract

**Background:**

The use of conventional foldable hydrophobic intraocular lenses (IOLs) in microincision cataract surgery (MICS) currently requires wound enlargement. We describe a combined surgical technique of MICS and trabeculectomy with insertion of a foldable IOL through the trabeculectomy fistula.

**Methods:**

After completion of MICS through two side port incisions, a 3.2 mm keratome is used to enter the anterior chamber under the previously outlined scleral flap. An Acrysof multi piece IOL (Alcon labs, Fort Worth, Tx) is inserted into the capsular bag through this incision. The scleral flap is then elevated and a 2 × 2 mm fistula made with a Kelly's punch. The scleral flap and conjunctival closure is performed as usual.

**Results:**

Five patients with primary open angle glaucoma with a visually significant cataract underwent the above mentioned procedure. An IOL was implated in the capsular bag in all cases with no intraperative complications. After surgery, all patients obtained a best corrected visual acuity of 20/20, IOL was well centered at 4 weeks follow up. The mean IOP (without any antiglaucoma medication) was 13.2 + 2.4 mm Hg at 12 weeks with a well formed diffuse filtering bleb in all the cases.

**Conclusion:**

The technique of combining MICS with trabeculectomy and insertion of a foldable IOL through the trabeculectomy fistula is a feasible and valuable technique for cases which require combined cataract and glaucoma surgery.

## Background

The combined surgical technique of phacotrabeculectomy has become a common technique for management of eyes with co-existent cataract and glaucoma [1,2]. Phacotrabeculectomy is either done as a single site surgery with both phacoemulsification and trabeculectomy performed from the same site or more commonly as a two-site surgery. Separating the two incisions may decrease the inflammation and subsequent fibrosis induced by the surgery leading to a better survival of the filtering bleb [2-4].

Microincision cataract surgery (MICS) or Phakonit (implying phacoemulsification performed with a needle) is a recently introduced bimanual technique that permits phacoemulsification via sub 1–1.2 mm incisions. The basic principle is to separate the irrigation from the phacoemulsification handpiece and use an irrigating chopper to maintain the anterior chamber. The advantages of MICS include an anastigmatic, safer, closed chamber surgery via smaller incisions and rapid visual restoration with minimal post operative inflammation [2,5-11]. We introduce a new technique of combining MICS with trabeculectomy and insertion of a foldable IOL through the trabeculectomy fistula.

## Methods

**Figure 1:**
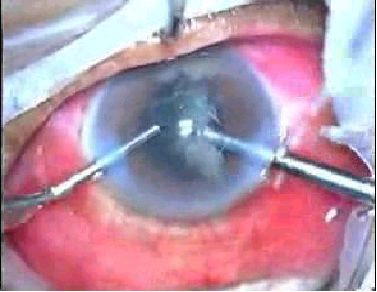
Bimanual microincision phacoemulsification being performed.

**Figure 2:**
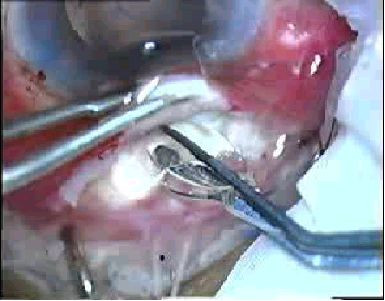
Acrysof multipiece piece IOL being introduced under the scleral flap.

**Figure 3:**
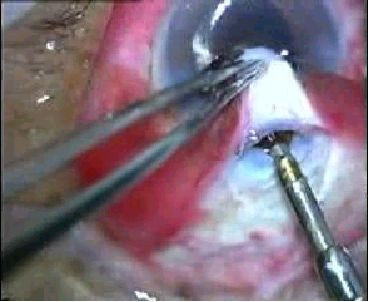
Trabeculectomy ostium being cut with the Kelly's descemet punch.

We performed MICS with trabeculectomy and insertion of a foldable IOL through the trabeculectomy fistula in 5 eyes of 5 patients in which combined cataract and glaucoma surgery was indicated due to significant cataract and medically uncontrolled IOP. Surgery was performed under peribulbar anesthesia. The eye was cleaned and draped and a superior rectus bridle suture was inserted. A limbus-based conjunctival flap was made, starting 10 mm behind the superior limbus. The conjunctiva was dissected to the limbus and a 4 × 4 mm rectangular scleral flap was fashioned in the superonasal quadrant. The flap was undermined and dissected to clear cornea using a steel crescent knife. The conjunctiva was then reposited back and cataract surgery was started. Two 1.2 mm clear-corneal side port incisions were made at 10 o'clock and 2 o'clock with a MVR knife. The anterior capsular staining with 0.1 cc trypan blue (0.06%) under air bubble was done in cases with poor or no red reflex. The anterior chamber was filled with 1% sodium hyaluronate. A bent 26G needle was used to perform capsulohexis and gentle hydrodissection was then performed. An irrigating chopper was inserted via the 2 o'clock port and then a sleeveless 20 G phaco tip was inserted through the 10 o'clock port. The infusion was maintained with two bottles of balanced salt solution connected together via a "Y" shaped tubing to increase the infusion (TURP tubing-trans uretheral resection of prostrate tubing) raised to 140 cm. Bimanual phacoemulsification (Figure 1) using stop and chop nucleotomy technique with the WhiteStar Sovereign phacoemulsification system (Advanced Medical Optics, Inc) was performed. The cortical matter was removed with bimanual irrigation aspiration system. After cortical removal, 1% sodium hyaluronate was injected into the anterior chamber. A 3.2 mm keratome was used to enter the anterior chamber under the previously outlined scleral flap. An Acrysof multi piece IOL (Alcon labs, Fort Worth, Tx) was inserted into the capsular bag through this incision using a holder and folder (Figure 2). The scleral flap was then elevated and a 2 × 2 mm fistula made with a Kelly's punch (Figure 3). An iridectomy was performed and the scleral flap was closed with two 10-0 monofilament sutures. Viscoelastic was removed from the anterior chamber and the capsular bag using bimanual irrigation aspiration via the two side ports. The side ports were hydrated and the conjunctiva closed with a running 8-0 vicryl suture. A subconjunctival injection of 0.1 cc dexamethasone was given and all patients received 1% prednisolone acetate eye drops (6 times/day), 0.3% ciprofloxacin eye drops (3 times/day) and 1% tropicamide (HS) in the post operative period.

## Results

Five patients with primary open-angle glaucoma with a visually significant cataract were operated with the above mentioned technique (Table 1). Pre-operatively all patients had an IOP > 21 mmHg on two or more topical anti-glaucoma medications. After surgery, visual acuity on the first post operative day ranged from 20/20 to 20/40. All patients obtained a best corrected visual acuity of 20/20 at 4 weeks follow up. The mean IOP (without any antiglaucoma medication) was 12.4 + 2.6 mm Hg at 4 weeks and 13.2 + 2.4 mm Hg at 12 weeks with a well- formed diffuse filtering bleb in all the cases.

**Table 1:** 

**Case No**	**Pre-op VA**	**Post-Op VA (4 weeks)**	**Pre-op Mean IOP (of diurnal curve)**	**Post-op Mean IOP (12 weeks)**	**Pre-op Antiglaucoma medications**	**Post-op Antiglaucoma medications**
1	20/200	20/20	24.5	15.6	Timolol, Latanoprost	Nil
2	20/120	20/20	27.8	11.0	Timolol, Pilocarpine, Brimonidine.	Nil
3	20/60	20/15	25.6	12.0	Latanoprost, Brimonidine	Nil
4	20/120	20/20	30.4	16.0	Timolol, Bimatoprost, Brimonidine	Nil
5	20/80	20/15	24	11.4	Timolol, Brimonidine, Dorzolamide	Nil

## Discussion

Combined surgery involving phacoemulsification and trabeculectomy has a reduced success rate compared to trabeculectomy alone [12]. This may be related to additional inflammation induced during cataract extraction [13]. Two-site surgery, separating the phacoemulsification and the trabeculectomy sites has theoretical advantages of reducing inflammation at the site of the filter and thereby may decrease the stimulus for the subsequent fibroblastic response [2-4]. Standard two site phacotrabeculectomy requires two large incisions, one for the cataract surgery and the other as the ostium under the scleral flap. In addition, the surgeon needs to adjust his position intraoperatively along with that of his assistants and equipments (i.e superior for trabeculectomy and temporal for phacoemulsification).

MICS/Phakonit offers the dual advantage of performing a bimanual closed chamber surgery and removing the cataract via an incision which is one-third the size of a routine phacoemulsification incision (1–1.2 mm versus a standard 3.2 mm incision) [6-8]. These ultra small incisions induce less inflammation [2,8-11] which is a significant advantage when this surgery is combined with a trabeculectomy. A reduction in post-operative inflammation is likely to reduce the risk of fibrosis and filter failure.

This technique offers the additional advantage of implantation of a standard hydrophobic acrylic IOLs with reduced risk of posterior capsule opacification [14] through the ostium of the trabeculectomy incision. One does not have to enlarge the side port incisions for introducing the IOL. Although new ultrathin IOLs (such as the ThinOptx rollable IOL) have been implanted after MICS, further investigation is required before they are adopted as a standard procedure in cataract surgery [15].

The combined two site surgery by this technique can be performed with surgeon sitting superiorly i.e without the need to change position intraoperatively. This considerably reduces the surgical time. This technique maintains the integrity of the small incisions used for cataract surgery and does not increase the inflammation that can be caused by a larger wound and additional manipulation. We believe thus that the combination of MICS with trabeculectomy is a valuable technique for management of eyes with co-existent cataract and glaucoma.

## Conclusion

The technique of combining MICS with trabeculectomy and insertion of a foldable IOL through the trabeculectomy fistula is a feasible and valuable technique for cases which require combined cataract and glaucoma surgery.

## Abbreviations

MICS : Micro-incision cataract surgery

IOL : Intraocular lens

## Competing interests

The author(s) declare that they have no competing interests.

## Authors' contributions

TD : Conceived the idea of above technique, and performed the surgeries.

RM : Helped in documentation of cases

HSS : Compiled the data and helped in the preparation of the manuscript

All authors read and approved the final manuscript.

## Pre-publication history

The pre-publication history for this paper can be accessed here:


